# Effect of feeding breeding pigeons pellets with different ring die compression ratio on their reproductive performance, and growth performance, meat quality and intestinal health of squab pigeons

**DOI:** 10.1016/j.psj.2026.106796

**Published:** 2026-03-14

**Authors:** Kang Cheng, Daizi Hu, Yuanqing Ma, Jinxiu Yao, Chenyu Chang, Hongyue Zhao, Minjun Yang, Jinrong Wang, Yong Zhang

**Affiliations:** a School of Biological Engineering, Henan University of Technology, Zhengzhou 450001, China; b School of International Education, Henan University of Technology, Zhengzhou 450001, China; c Henan Tiancheng Pigeon Industry Co., Ltd, Wugang 462500, China

**Keywords:** Pellets, Ring die compression ratio, Meat quality, Intestinal health, Pigeons

## Abstract

This study explore effect of feeding breeding pigeons pellets with different ring die compression ratio (RDCR) on their reproductive performance, and growth performance, meat quality and intestinal health of squab pigeons. A total of 150 pairs of White King breeding pigeons were randomly assigned to five groups and were fed pellets with a RDCR of 1:6, 1:6.5, 1:7, 1:7.5 or 1:8, respectively, for 46 days. Hardness and durability of pellets, as well as average daily feed intake of breeding pigeons during 1 to 46 days increased linearly with RDCR (*P* < 0.05). RDCR linearly and/or quadratically increased (*P* < 0.05) glucose level, as well as alanine aminotransferase and aspartate aminotransferase activities, and linearly reduced (*P* < 0.05) creatinine level in serum of squabs. RDCR decreased pH (quadratic, *P* < 0.05), drip loss (linear and/or quadratic, *P* < 0.05), and L*_48__h_ (linear and quadratic, *P* < 0.05) in pectorales. RDCR linearly and quadratically increased jejunal villus height, jejunal *ZO1* and ileal *ZO2* mRNA expression (*P* < 0.05). RDCR linearly increased a*_48__h_ and b*_48__h_ in pectorales, as well as ileal villus height and villus height to crypt depth ratio (*P* < 0.05), while linearly decreased ileal crypt depth (*P* < 0.05). Ileal total superoxide dismutase activity was enhanced quadratically by RDCR (*P* < 0.05). Overall, under the present experimental conditions, increasing the RDCR improved pellet quality, meat quality, intestinal morphology, redox status, and barrier function in squabs.

## Introduction

Pigeon meat is widely popular, especially in China, due to its low cholesterol, high protein, and distinctive flavor and aroma (Zhang et al., 2025). After forty years of development, pigeons have become the fourth most popular domestic poultry in China after chickens, ducks, and geese. In China, more than 10 billion squabs were consumed in 2025. This was from about 60 million pairs of breeding pigeons. Pellet compound feed is an important diet type for commercial or industrial pigeon farms. According to the majority of animal nutritionists and manufacturing specialists, poultry prefer to eat pellet compound feed in comparison to mash diets (Borovik et al., 2025). Pellets have a positive impact on poultry productivity. The increased growth rate and better feed conversion in poultry fed pellets can be attributed to the higher digestibility of nutrients in the feed and balanced nutrition (Lv et al., 2015; Zang et al., 2009). However, there are few and small-scale feed companies specializing in the production of meat pigeon pellets. In many cases, pigeon breeding companies prepare pellet feed themselves, making it difficult to ensure the quality of pellets. Pellets quality significantly influences the productivity enhancements of pigeons.

Pellets quality depends on the following factors: feed formulation, feed raw material quality and feed processing (Alsaftli, 2020). Consequently, the scientists made great efforts to improve the efficiency and economy of pigeon feeding by developing several methods of feed processing. In the processing technology, the quality of pellets is greatly affected when it passes through the pellet mill ring die (Amin and Sobhi, 2023). The productivity and quality of pellets vary among pellet mill with different ring die specifications. The ring die compression ratio (RDCR) is the ratio of the effective length to the diameter of the ring die hole, and reflects the extrusion strength of the hole wall on the granular feed inside it. When the diameter of the ring die hole is constant, the compression ratio is proportional to the effective length of the mold hole. A lower RDCR can reduce the quality and palatability of pellets, leading to inhibition of growth performance (Dai et al., 2011). High RDCR can inhibit the bioavailability of heat-sensitive nutrients such as vitamins and proteins, resulting in growth retardation (Wang et al., 2017). Moderate RDCR can decrease anti-nutritional factors and harmful microorganisms in the feed while meeting the pellet hardness requirements, reducing feed waste and improving digestion and utilization efficiency to promote growth (Wang et al., 2017). However, no report on effect of RDCR on pigeons’ pellets quality and performance is available. Maintaining an appropriate RDCR is necessary during pigeon feed processing. Therefore, the present experiment was designed to explore effect of RDCR on pellet quality, reproductive performance of breeding pigeons, growth performance, meat quality and intestinal function of squab pigeons, providing a theoretical foundation for scientific and rational utilization of ring die in the production of pigeon pellets.

## Materials and methods

### Birds and experimental design

The experiment was approved by the Henan University of Technology Institutional Animal Care and Use Committee. A total of 150 pairs of White King breeding pigeons, aged between 16 and 18 months and exhibiting similar performance, were selected and then randomly divided into five groups. Each treatment had six replicates and each replicate had five pairs of parent pigeons. The parent pigeons were fed a pellet feed with a RDCR of 1:6 (control group), 1:6.5, 1:7, 1:7.5 or 1:8 for 46 days, respectively. The levels of RDCR used in this investigation were chosen based on a previous study of broilers, which showed that feeding pellets with a RDCR of 1:6 exhibits superior growth performance, but feeding pellets with a RDCR of 1:8 begins to show a decrease in growth performance (Wang et al., 2017). Five kinds of pellets feed were produced by fixing the die hole diameter (3.0 mm) and varying the die hole length-radial ratio (6:1, 6.5:1, 7:1, 7.5:1, and 8:1). The feed formulation and pellet production process are consistent except for the RDCR. The composition and nutrient levels of the experimental diet are presented in Table S1. The pre-feeding period was 7 days. This experiment time frame included incubation (18 days) and lactation (28 days). During lactation period, one pair of parent pigeons with three squabs were reared in one cages equipped with a nest and a perch. During the entire experimental period, birds had free access to health sand and water. Parent pigeons were fed at regular intervals from 7:00 am, with ad libitum access to feed, and fasted every evening at 7:00 pm.

### Pellets hardness and pellet durability index (PDI)

Pellet hardness was determined using a sclerometer (ST120B, Jinan Shengtai Instrument Co., Ltd., Jinan, China). A PDI instrument (ST-136, Jinan Shengtai Instrument Co., Ltd., Jinan, China) to score the PDI.

### Reproductive performance, feed intake and final body weight (BW) of breeding pigeons

The fertilization rate, hatchability, laying interval, 38-day, 42-day, and 46-day laying rates, feed intake and final BW were determined according to our previous methods (Cheng et al., 2025).

### Growth performance of squab pigeons

At 1, 7, 14, 21, and 28 day of age, BW of the squab pigeons were weighed per cage. Average daily gain (ADG) was calculated for the days 1-7, days 8-14, days 15-21, days 22-28, and over all lactation periods (days 1-28), respectively.

### Sample collection of squab pigeons

At 28 days of age, two squabs per replicate were randomly selected and weighed. Blood samples were taken via the wing vein and then centrifuged at 4,000 rpm for 10 min to harvest serum. Serum was stored at −80°C until analysis. Then the birds were sacrificed by cervical dislocation. The left portion of breast meats (pectoralis major) were dissected (connective and adipose tissues removed) and stored at 4°C for meat quality measurement. Approximately 0.5-cm-long segments of the mid-jejunum and ileum were collected, flushed with physiological saline, and fixed in 4% polyformaldehyde to assess intestinal morphology.The jejunum and ileum mucosa samples were put into two sterile tubes, immediately snap-frozen in liquid nitrogen, and stored at −80°C for the determination of redox status and the gene expression.

### Determination of meat quality

The pH values were determined at 24 and 48 h postmortem using a hand-held pH meter (Testo-250, Testo SE & Co. KGaA, Lenzkirch, Germany) at three different locations per breast meat sample. Drip loss was determined as the previous study described (Zhang et al., 2015). Briefly, about 2 g meat was weighed, suspended by paper clips in the box that was covered by the plastic wrap, and stored at 4 °C. The meat samples were dried with filter paper and weighed at 24 and 48 h postmortem, respectively. Drip loss 24 h (%) = [(initial weight - weight 24 h)/initial weight] × 100; Drip loss 48 h (%) = [(initial weight - weight 48 h)/initial weight] × 100.

To measure the cooking loss, a small piece of breast muscle samples were weighed and then kept in a plastic bag. An 80 °C thermostatic water bath was performed until the internal temperature of the cooked meat samples were arrived at 70 °C. After cooling by running water, the samples were wiped off and reweighed. Cooking loss (%) was calculated as: [(initial weight-final weight)/initial weight] × 100. Meat color (L*=lightness, a*=redness, b*=yellowness) was detected at 24 and 48 h postmortem with a colorimeter (LS173, Shenzhen Linshang Technology Co., Ltd, Shenzhen, China), respectively.

### Serum parameters analysis

In squab pigeons, serum glucose, total protein, urea nitrogen, creatinine, low-density lipoprotein cholesterol, high-density lipoprotein cholesterol, total cholesterol and triglyceride levels as well as alkaline phosphatase, alanine transaminase (ALT) and aspartate transaminase (ALT) activities were analyzed using commercial kits (Nanjing Jiancheng Institute of Bioengineering, Nanjing, China).

### Intestinal morphology analysis

After fixation, intestinal samples were dehydrated, cleared, embedded, sectioned, stained, and photographed under a light microscope (RVL-100-G; ECHO Laboratories, San Diego, CA, USA). In each section, villus height (VH) and crypt depth (CD) were measured using Image-Pro Plus 6.0 software (Media Cybernetics, San Diego, CA, USA), and then calculated VH to CD ratio.

### Intestinal redox status assay

The redox status of intestine was reflected by the malondialdehyde (MDA), total superoxide dismutase (T-SOD), catalase (CAT) and glutathione peroxidase (GPX). The MDA content, T-SOD, GPX and CAT activities were determined using commercial kits purchased from Nanjing Jiancheng Institute of Bioengineering (Nanjing, China). The protein concentration of intestinal homogenates was measured using a bicinchoninic acid assay kit (Nanjing Jiancheng Institute of Bioengineering, Nanjing, China) to adjust the results of intestinal redox status.

### Intestinal gene expression analysis

Total RNA was extracted from intestinal sample of squab pigeons using the TRIzol reagent (Vazyme Biotech Co., Ltd., Nanjing, China). The RNA concentration, quality and reverse transcription of each sample as well as the mRNA relative expression of target genes were performed as previously described (Cheng et al., 2024, 2023b). Glyceraldehyde-3-phosphate dehydrogenase and beta actin were used as the internal reference gene to normalize the mRNA expression levels of the target genes. The primer sequences used in this trail are shown in Table S2. The relative expressions of the target genes were calculated with the 2^-ΔΔCt^ method.

### Statistical analysis

Data were analyzed by using SPSS statistical software (ver. 22.0 for Windows, SPSS Inc., Chicago, USA). Differences among groups were determined via one-way analysis of variance and Tukey’s post hoc test for multiple comparisons when *F* was significant. Orthogonal polynomial contrasts were used to assess the linear and quadratic effects of increasing levels of RDCR. Statistical significance level was set at *P* < 0.05. Results were displayed as means with their standard deviations.

## Results

### Pellets hardness and PDI

**Fig. 1:**
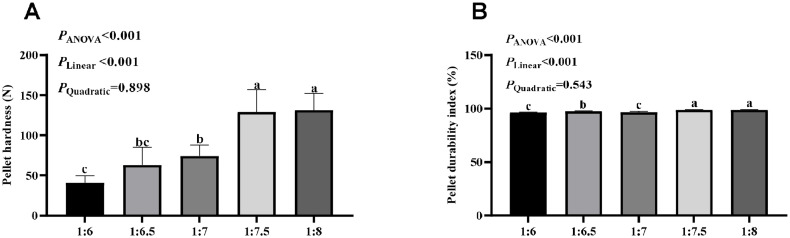
Effect of different ring die compression ratio on the pellet hardness (A) and pellet durability index (PDI, B) of pellet feeds. Means without a common letter differ, *P* < 0.05. Results are expressed as means and standard deviations.

As presented in the Fig. 1, RDCR linearly increased pellets hardness (*P* < 0.001) and PDI (*P* < 0.001). The 1:7.5 and 1:8 RDCR groups had higher pellets hardness and PDI than the other three groups (*P* < 0.05). Pellets hardness in the 1:6 group was lower than the 1:7 RDCR group (*P* < 0.05), and its PDI was lower than the 1:6.5 RDCR group (*P* < 0.05).

### Reproductive performance, feed intake and final BW of parent pigeons

No significant differences in 38-day laying rate, 42-day laying rate, 46-day laying rate, fertility rate, hatchability, laying interval and final BW of parent pigeons (Table 1) were observed among treatments (*P* > 0.05). During the entire experimental periods, average daily feed intake (ADFI) of parent pigeons increased linearly with increasing RDCR (*P* < 0.001). ADFI in the 1:7.5 RDCR group was higher than the 1:6, 1:6.5 and 1:7 groups (*P* < 0.05). Compared with the 1:6.5 RDCR group, the 1:8 group exhibited a higher ADFI (*P* < 0.05).

**Table 1:** Effect of pellet feed with different ring die compression ratio on the reproductive performance, feed intake and final body weight of breeding pigeons.

Items	Ring die compression ratio					*P*_ANOVA_	*P*_Linear_	*P*_Quadratic_
	1:6	1:6.5	1:7	1:7.5	1:8			
38-day laying rate (%)	53.57±24.69	64.26±21.51	80.82±19.01	83.89±15.38	74.01±23.14	0.110	0.035	0.101
42-day laying rate (%)	92.36±8.51	87.44±11.23	89.22±13.17	91.81±9.29	89.75±11.77	0.935	0.952	0.699
46-day laying rate (%)	100.00±0.00	96.67±8.16	100.00±0.00	97.92±5.10	100.00±0.00	0.552	0.824	0.418
Fertility rate (%)	81.96±22.07	82.44±18.47	78.92±10.89	72.96±29.25	79.54±24.82	0.948	0.618	0.774
Hatchability (%)	97.92±5.10	86.01±18.09	98.15±4.54	92.50±8.66	93.75±10.46	0.290	0.893	0.602
Laying interval (d)	34.91±1.97	33.47±1.73	34.18±2.01	35.34±1.76	35.60±1.81	0.291	0.190	0.188
Average daily feed intake (1-46 d, g/pair/d)	144.57±9.14^bc^	134.81±13.09^c^	151.10±8.45^bc^	169.61±11.03^a^	156.93±10.59^ab^	<0.001	<0.001	0.824
Final body weight (g/pair)	1104.59±29.60	1114.88±56.38	1109.02±70.78	1156.88±49.34	1110.23±37.18	0.393	0.424	0.445

Different lowercase letters indicate statistical significance (*P* < 0.05). Results are expressed as means and standard deviations.

### Growth performance of squab pigeons

In Table 2, RDCR did not alter BW at 7, 14, 21 and 28 days of age, or ADG during the days 1-7 period, 8-14 period, 15-21 period, 22-28 period and 1-28 period of squab pigeons (*P* > 0.05).

**Table 2:** Effect of pellet feed with different ring die compression ratio on the growth performance of squabs.

Items	Ring die compression ratio					*P*_ANOVA_	*P*_Linear_			*P*_Quadratic_
	1:6	1:6.5	1:7	1:7.5	1:8					
Body weight (g/bird)										
1 d	15.98±0.19	15.98±0.15	15.97±0.16	15.94±0.21	15.95±0.09	0.991		0.685	0.992	
7 d	147.57±22.03	141.54±17.55	135.85±14.87	141.93±14.77	118.17±21.50	0.090		0.021	0.408	
14 d	355.53±24.21	357.12±26.00	369.81±29.86	374.56±23.76	344.60±22.37	0.284		0.894	0.079	
21 d	463.37±24.01	466.59±18.30	475.07±11.81	451.12±21.48	464.28±18.20	0.334		0.587	0.672	
28 d	483.35±23.61	487.81±19.52	497.79±17.57	491.42±34.84	507.87±13.44	0.409		0.088	0.830	
Average daily gain (g/bird/d)										
1-7 d	18.80±3.16	17.94±2.52	17.13±2.12	18.00±2.12	14.60±3.08	0.092		0.022	0.408	
8-14 d	29.71±2.39	30.80±2.33	33.42±3.11	33.23±2.99	32.35±2.11	0.091		0.031	0.103	
15-21 d	15.41±3.79	15.64±2.23	15.04±3.74	10.94±3.72	17.10±4.05	0.067		0.777	0.138	
22-28 d	2.86±1.48	3.03±0.90	3.25±1.37	5.76±3.71	6.23±3.17	0.100		0.005	0.438	
1-28 d	16.69±0.85	16.85±0.70	17.21±0.62	16.98±1.24	17.57±0.48	0.406		0.087	0.830	

Results are expressed as means and standard deviations.

### Serum parameters in squab pigeons

As shown in Table 3, RDCR increased glucose (linear, *P* < 0.001; quadratic, *P* = 0.038) content, as well as ALT (linear, *P* < 0.001; quadratic, *P* < 0.001) and AST (quadratic, *P* < 0.001) activities, and reduced creatinine (linear, *P* < 0.001) level in serum. Serum glucose levels in the 1:7.5 and 1:8 RDCR groups were higher than another three groups (*P* < 0.05). The 1:7, 1:7.5 and 1:8 RDCR groups showed a lower creatinine level compared with the 1:6 RDCR group, and the values of this parameter in the 1:7.5 and 1:8 RDCR groups were lower than the 1:1:6.5 group (*P* < 0.05). AST activity in the 1:7 RDCR group and ALT activity in the 1:6.5 RDCR group were higher than the 1:6, 1:7.5 and 1:8 RDCR groups (*P* < 0.05). Serum total protein, urea nitrogen, low-density lipoprotein cholesterol, high-density lipoprotein cholesterol, total cholesterol and triglyceride levels as well as alkaline phosphatase activity were not affected (*P* > 0.05).

**Table 3:** Effect of pellet feed with different ring die compression ratio on the serum parameters in squabs.

Items	Ring die compression ratio					*P*_ANOVA_	*P*_Linear_	*P*_Quadratic_
	1:6	1:6.5	1:7	1:7.5	1:8			
Glucose (mmol/L)	14.02±5.11^b^	12.69±3.87^b^	13.93±3.81^b^	18.53±2.50^a^	19.86±3.20^a^	<0.001	<0.001	0.038
Total protein (g/L)	26.11±5.05	24.94±4.73	27.96±5.24	30.37±5.71	28.61±5.85	0.121	0.036	0.757
Urea nitrogen (mmol/L)	1.55±1.83	1.61±1.00	2.06±1.16	2.36±0.88	2.15±0.71	0.385	0.076	0.595
Creatinine (μmol/L)	33.39±9.42^a^	23.54±12.65^ab^	20.99±9.27^bc^	11.64±9.30^cd^	7.70±6.57^d^	<0.001	<0.001	0.631
Aspartate aminotransferase (U/L)	1.96±1.22^b^	3.06±1.68^ab^	4.27±1.53^a^	2.53±1.36^b^	2.22±1.52^b^	0.003	0.987	<0.001
Alanine aminotransferase (U/L)	2.89±1.16^bc^	5.21±1.35^a^	3.67±2.05^ab^	2.96±1.58^bc^	1.55±1.14^c^	<0.001	<0.001	<0.001
Low-density lipoprotein cholesterol (mmol/L)	1.86±1.35	1.46±0.49	1.86±0.95	1.75±0.59	1.53±0.66	0.688	0.635	0.868
High-density lipoprotein cholesterol (mmol/L)	7.70±2.88	7.86±3.11	5.83±1.57	7.16±2.80	6.75±2.14	0.311	0.272	0.427
Total cholesterol (mmol/L)	5.81±0.93	6.07±1.81	5.19±1.72	5.16±1.17	5.46±1.56	0.493	0.238	0.558
Triglyceride (mmol/L)	1.22±0.50	0.86±0.26	1.12±0.48	0.90±0.32	0.87±0.23	0.064	0.056	0.662
Alkaline phosphatase (U/L)	205.49±113.71	209.73±170.48	202.76±95.09	275.60±136.07	224.53±113.26	0.618	0.379	0.825

Different lowercase letters indicate statistical significance (*P* < 0.05). Results are expressed as means and standard deviations.

### Meat quality in squab pigeons

RDCR of 1:7 and 1:7.5 quadratically (*P* = 0.003) reduced pH at 24 h (Table 4). RDCR of 1:7 quadratically (*P* < 0.001) reduced pH at 48 h. RDCR of 1:7 and 1:7.5 linearly (*P* = 0.021) and quadratically (*P* < 0.001) reduced L* at 48 h. RDCR of 1:8 linearly increased a* and b* at 48 h (*P* < 0.001). RDCR of 1:7 to 1:8 quadratically reduced drip loss at 48 h (*P* < 0.001), while RDCR of 1:6.5 to 1:8 linearly and quadratically reduced drip loss at 24 h (*P* < 0.001). L*, a* and b* at 24 h, as well as cooking loss were not altered by the RDCR levels (*P* > 0.05).

**Table 4:** Effect of pellet feed with different ring die compression ratio on the meat quality in squabs.

Items	Ring die compression ratio					*P*_ANOVA_	*P*_Linear_	*P*_Quadratic_
	1:6	1:6.5	1:7	1:7.5	1:8			
Cooking loss (%)	39.99±6.67	37.87±4.74	39.72±6.81	38.73±3.98	36.67±2.92	0.520	0.231	0.633
24 h after slaughter								
pH	6.32±0.26^a^	6.08±0.33^ab^	5.91±0.22^b^	5.84±0.34^b^	6.25±0.75^ab^	0.029	0.328	0.003
L*	44.11±3.16	42.01±3.06	44.65±5.23	44.60±2.16	46.24±3.14	0.073	0.036	0.208
a*	12.62±1.79	11.86±2.35	12.46±2.20	11.57±2.34	12.41±2.36	0.746	0.728	0.477
b*	7.03±2.04	6.14±1.85	7.07±1.52	5.84±1.52	6.80±2.56	0.426	0.669	0.464
Drip loss (%)	39.22±9.86^a^	25.08±10.89^b^	18.48±5.37^bc^	16.56±4.13^c^	14.16±2.66^c^	<0.001	<0.001	<0.001
48 h after slaughter								
pH	5.93±0.14^a^	5.80±0.16^a^	5.59±0.13^b^	5.84±0.09^a^	5.85±0.18^a^	<0.001	0.392	<0.001
L*	44.03±2.47^a^	41.50±2.85^ab^	39.96±2.70^b^	40.92±2.22^b^	41.71±1.56^ab^	0.002	0.021	<0.001
a*	10.83±1.59^c^	12.09±2.41^bc^	12.39±1.68^bc^	13.44±2.34^ab^	15.44±1.70^a^	<0.001	<0.001	0.299
b*	5.49±1.72^b^	5.31±2.16^b^	6.16±2.36^ab^	6.96±2.76^ab^	8.45±2.10^a^	0.007	<0.001	0.179
Drip loss (%)	50.24±10.74^a^	41.80±12.30^a^	29.62±7.89^b^	29.25±6.82^b^	27.88±7.44^b^	<0.001	0.080	<0.001

Different lowercase letters indicate statistical significance (*P* < 0.05). Results are expressed as means and standard deviations.

### Intestinal morphology in squab pigeons

As shown in the Table 5, RDCR increased intestinal VH (jejunum: linear, *P* = 0.001; quadratic, *P* = 0.004; ileum: linear, *P* < 0.001) and ileal VH: CD (linear, *P* < 0.001) and decreased ileal CD (linear, *P* < 0.001). Compared with the 1:6 and 1:6.5 RDCR groups, increased ileal VH and VH:CD were found in the 1:7 to 1:8 RDCR groups (*P* < 0.05), a higher jejunal VH was observed in the 1:7 and 1:7.5 RDCR groups (*P* < 0.05), and a lower ileal CD was seen in the 1:8 RDCR group (*P* < 0.05). Moreover, ileal VH:CD in the 1:8 RDCR group was higher than the 1:7.5 RDCR group (*P* < 0.05). Jejunal VH:CD and CD in squab pigeons were not changed (*P* > 0.05).

**Table 5:** Effect of pellet feed with different ring die compression ratio on the intestinal morphology in squabs.

Items	Ring die compression ratio					*P*_ANOVA_	*P*_Linear_	*P*_Quadratic_
	1:6	1:6.5	1:7	1:7.5	1:8			
Jejunum								
Villus height (μm)	657.03±96.04^b^	670.51±101.51^b^	827.61±180.72^a^	844.39±94.29^a^	742.97±93.22^ab^	<0.001	0.001	0.004
Crypt depth (μm)	170.05±51.35	138.26±29.94	160.39±41.50	184.69±59.10	138.60±29.48	0.184	0.670	0.564
Villus height to crypt depth ratio	4.31±1.76	5.01±1.13	5.29±1.21	4.93±1.28	5.53±1.04	0.182	0.043	0.536
Ileum								
Villus height (μm)	629.71±69.95^b^	632.21±49.54^b^	750.14±73.85^a^	750.76±114.19^a^	788.18±44.52^a^	<0.001	<0.001	0.542
Crypt depth (μm)	154.49±34.13^a^	154.24±39.96^a^	125.28±33.48^ab^	130.73±20.65^ab^	107.64±15.91^b^	<0.001	<0.001	0.721
Villus height to crypt depth ratio	4.26±1.05^c^	4.39±1.33^c^	6.25±1.21^ab^	5.81±0.91^b^	7.46±1.08^a^	<0.001	<0.001	0.527

Different lowercase letters indicate statistical significance (*P* < 0.05). Results are expressed as means and standard deviations.

### Intestinal redox status in squab pigeons

RDCR of 1:8 quadratically (*P* = 0.040) increased ileal T-SOD activity in squab pigeons compared to the 1:7 RDCR group (Table 6). No significant difference in intestinal MDA level, CAT and GPX activities as well as jejunal T-SOD activity was seen (*P* > 0.05).

**Table 6:** Effect of pellet feed with different ring die compression ratio on the intestinal redox status in squabs.

Items	Ring die compression ratio					*P*_ANOVA_	*P*_Linear_	*P*_Quadratic_
	1:6	1:6.5	1:7	1:7.5	1:8			
Jejunum								
Malondialdehyde (nmol/mg protein)	0.75±0.67	0.70±0.60	0.95±0.57	1.07±0.68	0.71±0.50	0.781	0.718	0.423
Catalase (U/mg protein)	85.43±13.73	84.55±21.85	81.58±15.98	79.55±24.72	90.07±12.61	0.884	0.858	0.406
Total superoxide dismutase (U/mg protein)	218.78±50.78	205.97±56.09	207.51±52.43	214.55±64.46	247.04±31.81	0.660	0.344	0.240
Glutathione peroxidase (U/mg protein)	120.26±37.67	123.34±67.10	120.25±43.69	103.52±53.26	149.12±73.37	0.734	0.609	0.417
Ileum								
Malondialdehyde (nmol/mg protein)	1.16±0.97	1.23±0.58	1.04±0.60	1.69±1.90	0.86±0.31	0.709	0.914	0.553
Catalase (U/mg protein)	77.64±9.82	81.55±15.70	70.22±8.74	84.15±21.30	84.53±13.45	0.426	0.391	0.420
Total superoxide dismutase (U/mg protein)	171.60±10.60^ab^	180.01±25.61^ab^	161.97±22.79^b^	173.05±25.46^ab^	205.65±28.46^a^	0.039	0.054	0.040
Glutathione peroxidase (U/mg protein)	152.54±50.19	101.76±27.00	122.83±52.66	150.61±72.88	178.27±47.25	0.137	0.148	0.050

Different lowercase letters indicate statistical significance (*P* < 0.05). Results are expressed as means and standard deviations.

### Intestinal gene expression in squab pigeons

In Table 7, RDCR of 1:8 linearly (*P* = 0.005) increased jejunal *CAT* gene expression compared to the 1:6 RDCR group, linearly (*P* = 0.013) and quadratically (*P* = 0.040) upregulated jejunal *ZO1* mRNA expression compared to the 1:6, 1:6.5, and 1:7.5 RDCR groups, quadratically (*P* = 0.007) enhanced ileal *SOD2* gene expression compared to the 1:7.5 RDCR group, and linearly (*P* < 0.001) and quadratically (*P* = 0.010) upregulated ileal *ZO2* mRNA expression compared to other groups. Additionally, ileal *CLDN2* mRNA expression was linearly (*P* = 0.023) suppressed by 1:7.5 RDCR compared to the 1:6.5 RDCR group. There were no significant differences in jejunal *CLDN2, ZO2, CLDN1, SOD1, GPX2, GPX4* and *SOD2* mRNA expression as well as ileal *ZO1, CLDN1, CAT, GPX2, GPX4* and *SOD1* mRNA expression among treatments (*P* > 0.05).

**Table 7:** Effect of pellet feed with different ring die compression ratio on the intestinal genes expression related to barrier and antioxidant functions in squabs.

Items	Ring die compression ratio					*P*_ANOVA_	*P*_Linear_	*P*_Quadratic_
	1:6	1:6.5	1:7	1:7.5	1:8			
Jejunum								
*ZO1*	1.00±0.35^b^	0.84±0.45^b^	1.37±0.90^ab^	0.70±0.22^b^	2.46±1.43^a^	0.006	0.013	0.040
*ZO2*	1.00±1.21	1.14±1.67	1.40±1.60	1.97±0.86	2.61±1.06	0.225	0.025	0.518
*CLDN1*	1.00±0.27	1.16±0.83	2.30±1.95	0.98±0.38	3.70±3.09	0.188	0.024	0.315
*CLDN2*	1.00±0.24	1.24±1.11	1.02±0.45	1.11±0.63	1.08±0.40	0.970	0.966	0.815
*CAT*	1.00±0.62^b^	1.22±1.06^ab^	1.44±1.01^ab^	1.57±1.13^ab^	2.79±1.06^a^	0.040	0.005	0.221
*GPX2*	1.00±0.76	0.71±0.58	0.95±0.44	1.72±1.27	1.84±1.22	0.161	0.032	0.346
*GPX4*	1.00±0.34	0.56±0.30	1.05±0.58	0.70±0.26	1.20±0.51	0.072	0.319	0.108
*SOD1*	1.00±0.57	0.71±0.33	0.86±0.44	0.72±0.08	1.44±0.59	0.098	0.132	0.017
*SOD2*	1.00±0.43	0.69±0.36	0.80±0.35	0.86±0.33	1.19±0.70	0.393	0.363	0.093
Ileum								
*ZO1*	1.00±0.37	0.83±0.62	1.28±0.99	0.98±0.43	1.77±0.99	0.225	0.089	0.314
*ZO2*	1.00±0.28^b^	0.89±0.14^b^	1.32±0.42^b^	0.81±0.40^b^	2.05±0.64^a^	<0.001	<0.001	0.010
*CLDN1*	1.00±0.20	1.12±1.27	0.96±0.36	1.02±0.36	1.44±0.31	0.684	0.344	0.404
*CLDN2*	1.00±0.36^ab^	1.43±0.60^a^	0.77±0.62^ab^	0.58±0.18^b^	0.74±0.22^ab^	0.024	0.023	0.917
*CAT*	1.00±1.58	0.98±0.48	0.98±0.31	0.85±0.56	1.45±0.68	0.771	0.489	0.398
*GPX2*	1.00±0.29	0.97±0.34	1.02±0.46	0.97±0.48	1.15±0.51	0.949	0.595	0.634
*GPX4*	1.00±0.42	0.88±0.60	0.85±0.26	0.82±0.16	1.14±0.39	0.625	0.684	0.161
*SOD1*	1.00±0.22	0.97±0.80	0.72±0.33	0.61±0.14	1.02±0.56	0.465	0.599	0.173
*SOD2*	1.00±0.25^ab^	0.94±0.22^ab^	0.75±0.19^ab^	0.72±0.28^b^	1.23±0.44^a^	0.033	0.524	0.007

Different lowercase letters indicate statistical significance (*P* < 0.05). *ZO1*, zonula occludens 1; *ZO2*, zonula occludens 2; *CLDN1*, claudin 1; *CLDN2*, claudin 2; *CAT*, catalase; *GPX2*, glutathione peroxidase 2; *GPX4*, glutathione peroxidase 4; *SOD1*, total superoxide dismutase 1; *SOD2*, total superoxide dismutase 2. Results are expressed as means and standard deviations.

## Discussion

In the current study, PDI and pellet hardness were improved by the increasing RDCR, with the greatest effects observed at the ratio of 1:7.5 and 1:8. Similar findings in broilers were found by Wang et al. (2017), who reported that pellet hardness increased in corn-, soybean meal- and rapeseed meal- based diet as the RDCR increased from 1:6 to 1:10. It was also found that rabbit pellet with a RDCR of 1:14 had higher PDI and hardness than pellets with RDCR from 1:6 to 1:12 (Chen, 2016). The improvements in pellet quality observed as the RDCR increased can be attributed to the increased extrusion time of feed raw materials in the ring hole, which enhances adhesion among the pellet components. In this study, RDCR especially at a the ratio of 1:7.5 linearly increased feed intake of parent pigeons. Additionally, it should be noted that there is no difference in feed intake between the 1:7.5 and 1:8 RDCR groups. The observed effects in squabs at different RDCR levels may be due to pellet physical properties affecting the parent pigeons' intake and digestion.

In a fasting state, plasma glucose concentrations are relatively stable, indicating that glucose production and utilization are equal (Giugliano et al., 2008). Serum creatinine concentration is commonly used as an indicator of renal function and as a measure of the glomerular filtration rate (Perrone et al., 1992). Changes in ALT and AST activities in serum can reflect liver homeostasis (Zhang et al., 2022). This study showed that increasing RDCR linearly and quadratically increased glucose level, and linearly reduced creatinine content in the serum of squabs, with these effects being more pronounced in 1:8 RDCR group. Increasing RDCR may elevate glucose level in serum of pigeons by enhancing intestinal digestion and absorption function. Although increasing RDCR quadratically increased AST and ALT activities in serum, we are unsure whether the levels of AST and ALT are within the normal physiological range. The 1:7 and 1:6.5 RDCR groups exhibited higher AST and ALT activities, respectively, indicating that 1:6.5 and 1:7 RDCR may cause metabolic fluctuations. Further research is needed to confirm the impact of RDCR on the function of pigeon livers and kidneys. However, scarce information is available regarding the effect of RDCR on blood metabolites in poultry.

Meat quality indicators such as drip loss, color, and cooking loss influence consumers' purchasing preferences (Pu et al., 2025). In this study, we found that increasing RDCR, particularly at a ratio of 1:8, decreased the drip loss of breast meat in a linear and/or quadratic manner. Moreover, pigeons fed pellets with increasing RDCR not only had redder meat, but also had lighter and yellower meat. The presence of free radicals before and after slaughter causes myofibrils protein denaturation and myofibrils shrank, which leads to the increased drip loss of meat (Zhang et al., 2011). Meat discoloration might be associated with metmyoglobin accumulation induced by free radicals (Faustman et al., 2010; Zhang et al., 2011). In addition, RDCR of 1:7 or 1:7.5 quadratically reduced pH of breast meat. The pH is a vital parameter reflecting the degradation speed of muscle glycogen after slaughter. This process is closely associated with color grading and drip loss (Cong et al., 2017). We assumed that the improved meat color and drip loss may be related to the redox balance in pigeons fed pellets with increasing RDCR. However, few studies have demonstrated the effects of RDCR on breast meat quality and its possible mechanisms.

The intestine plays a vital role in digesting and absorbing of nutrients. The morphological indicators of the intestine (VH, CD, and VH/CD) directly reflect an animals’ digestive capacity and intestinal health. However, studies on the effects of pellets with different RDCR on intestine morphology in poultry are lacking. In this study, the increased intestinal VH/CD as well as decreased ileal CD with increasing RDCR indicated enhanced digestive capacity. Previous study showed that the choice feeding of corn, wheat, and pellet feed exert a different response of intestinal morphology in pigeons, which may be attributed to the form and composition of diet (Liu et al., 2023). Broilers fed pellet-coarse diets had lower CD in the jejunum (Mohammadi Ghasem Abadi et al., 2019), and an increase in VH was observed in broilers fed a pellet diet (Naderinejad et al., 2016). Therefore, different RDCR may bring a different intake of dietary nutrients and forms, which in turn affects intestinal morphology in squab pigeons.

Under normal circumstances, an internal dynamic equilibrium exists between reactive oxygen species production and antioxidant defense systems (Cheng et al., 2023b). SOD as an endogenous cell defense mechanism plays an important role in mediating the detoxification of superoxide anion (Islam et al., 2022). The increased SOD activity improves the antioxidant capacity, which in turn enhances the ability to resist exogenous stimuli. Few studies have focused on the effects of RDCR on redox status in poultry. In the present study, RDCR quadratically increased ileal T-SOD activity in squab pigeons, with its moderate level being 1:8, which may be associated with the up-regulated *SOD2* transcription expression. These results indicated that pellets with different RDCR could affect intestinal redox status in pigeons.

Tight junction (TJ) proteins such as OCLN, ZO and CLDN build a physical barrier and allow selective paracellular permeability between the host and the external environment by coordinating with intestinal epithelial cells (Cheng et al., 2023a). TJ proteins expression and localization can undergo constant remodeling in response to various external stimuli (Arumugam et al., 2025). Dysregulation of TJ proteins that compromises barrier homeostasis is a contributor to intestinal injury and growth obstruction in pigeons (Xu et al., 2024). In the current study, RDCR, especially at a ratio of 1:8, increased the expression of jejunal *ZO1* and *CLDN1*, ileal *CLDN1*, and intestinal *ZO2* mRNA in a linear and/or quadratic manner. Moreover, RDCR linearly suppressed mRNA expression of ileal *CLDN2*, which plays a crucial role in mediating high permeability for cations and water (Wu et al., 2025). These results suggest that optimal RDCR could improve intestinal barrier function and permeability, and contribute to health status of squab pigeons. No report on the effect of RDCR on intestinal TJ proteins expression in poultry is available.

## Conclusion

In conclusion, increasing RDCR improves pellet quality, meat quality, intestinal morphology, redox status, and barrier function in squab pigeons. Based on our research findings, we recommend that the RDCR for the pellet feed of pigeons could be 1:8. However, further research is warranted to determine whether a larger RDCR is suitable for pigeons.

## Funding

This study was supported by the National Key Research and Development Program of China (grant number 2021YFD1300300).

## CRediT authorship contribution statement

**Kang Cheng:** Writing – original draft, Validation, Methodology, Investigation. **Daizi Hu:** Formal analysis. **Yuanqing Ma:** Formal analysis. **Jinxiu Yao:** Formal analysis. **Chenyu Chang:** Formal analysis. **Hongyue Zhao:** Resources. **Minjun Yang:** Resources. **Jinrong Wang:** Writing – review & editing, Supervision, Resources, Project administration, Funding acquisition, Data curation, Conceptualization. **Yong Zhang:** Conceptualization.

## Disclosures

The authors declare that they have no conflict of interest.

## Appendix Supplementary materials

Image, application 1

## Footnotes

Supplementary material associated with this article can be found, in the online version, at doi:10.1016/j.psj.2026.106796.
