# Supplementary material for: Effect of feeding breeding pigeons pellets with different ring die compression ratio on their reproductive performance, and growth performance, meat quality and intestinal health of squab pigeons
Source: Poult Sci. 2026 Mar 14;105(8):106796. doi: 10.1016/j.psj.2026.106796 (PMC13147296; doi:10.1016/j.psj.2026.106796)
Supplement: Supplementary file 1 [file mmc1.docx]

Table S1. Ingredients and nutrient composition of the experimental diets.

| Items | 1:6 | 1:6.5 | 1:7 | 1:7.5 | 1:8 |
| --- | --- | --- | --- | --- | --- |
| Ingredients (%) |  |  |  |  |  |
| Corn | 52.30 | 52.30 | 52.30 | 52.30 | 52.30 |
| Soybean meal | 18.30 | 18.30 | 18.30 | 18.30 | 18.30 |
| Peas | 18.75 | 18.75 | 18.75 | 18.75 | 18.75 |
| Sorghum | 3.75 | 3.75 | 3.75 | 3.75 | 3.75 |
| Soybean oil | 1.325 | 1.325 | 1.325 | 1.325 | 1.325 |
| Dicalcium phosphate | 1.555 | 1.555 | 1.555 | 1.555 | 1.555 |
| Limestone | 2.13 | 2.13 | 2.13 | 2.13 | 2.13 |
| L-Lysine | 0.07 | 0.07 | 0.07 | 0.07 | 0.07 |
| DL-Methionine | 0.11 | 0.11 | 0.11 | 0.11 | 0.11 |
| Premix^1^ | 1.00 | 1.00 | 1.00 | 1.00 | 1.00 |
| Sodium chloride | 0.30 | 0.30 | 0.30 | 0.30 | 0.30 |
| Zeolite powder | 0.41 | 0.41 | 0.41 | 0.41 | 0.41 |
| Total | 100.00 | 100.00 | 100.00 | 100.00 | 100.00 |
| Calculated nutrient levels^2^ |  |  |  |  |  |
| Crude protein | 17.15 | 17.06 | 17.19 | 17.05 | 17.15 |
| Metabolic energy (MJ/kg) | 12.03 | 12.03 | 12.03 | 12.03 | 12.03 |
| Calcium | 1.12 | 1.06 | 1.07 | 1.11 | 1.00 |
| Total phosphorous | 0.63 | 0.61 | 0.65 | 0.60 | 0.63 |
| Lysine | 0.96 | 0.96 | 0.96 | 0.96 | 0.96 |
| Methionine | 0.35 | 0.35 | 0.35 | 0.35 | 0.35 |

^1^Premix provided per kilogram of feed: vitamin A, 4000.00 IU; vitamin D3, 1725.00 IU; vitamin E, 24.00 mg; vitamin K3, 1.0 mg; vitamin B12,25.00 mg; vitamin B1, 3.0 mg; riboflavin, 13.0 mg; niacin, 15.00 mg; choline chloride, 200.0 mg; pantothenic acid, 7.50 mg; vitamin B6, 2.00 mg; biotin, 0.12 mg; folic acid, 0.55 mg; Fe, 35.0 mg; Cu, 10.0 mg; Mn, 55.0 mg; Zn, 35.0 mg; I, 0.20 mg; Se, 0.25 mg.

^2^Crude protein, calcium, and total phosphorous levels are analyzed values.

Table S2. The primer sequences used in this trail

| Genes | Forward primer (5'-3') | Reverse primer (5'-3') |
| --- | --- | --- |
| *CAT* | TGTGTGTCACGGGAGATGTG | TGTAGAACATCCGCACCTGG |
| *GPX2* | TGAATTATTGAGGCAGGGGGA | GCTGGTTGAGCTGGGTGTAA |
| *GPX4* | GCACCTTGGGCAATGCAATAA | ATGGGACTGTACCGCTTCAC |
| *SOD1* | AGGTGCTCACTTCAACCCTG | GGTCTCCCACATGCCTTTCT |
| *SOD2* | GAGGAGGAGAGCCTAAAGGAGA | TGTTATAGCCAAGCCACCCC |
| *ZO*1 | CCTTTGGACAAAGAGAAAGGTGA | CCAGGTTTTGGGGTCACAGT |
| *ZO2* | CCAGCCCTGAAGATGAAGCA | CAGCTGAGGTGCCTTCTTGA |
| *CLDN1* | GGTGGGGTGATGTTCATCGT | AGCCACTCTGTTGCCATACC |
| *CLDN2* | TTACCTGGGCATCACCTCCT | TCTTCTGTGTCTGGCTGACG |
| *β-actin* | GTTGCCCTGGATTTTGAGCA | CCAACAGATTCCATACCCAAGA |
| *GAPDH* | GTCAGCAATGCCTCTTGCAC | TGGCATGGACAGTGGTCATA |

*β-*-actin, beta actin; *CAT,* catalase; *CLDN1*, claudin 1; *CLDN2*, claudin 2; *GAPDH*, glyceraldehyde-3-phosphate dehydrogenase; *GPX2*, glutathione peroxidase 2; *GPX4*, glutathione peroxidase 4; *SOD1*, superoxide dismutase 1; *SOD2*, superoxide dismutase 2 ; *ZO1*, zonula occludens 1; *ZO2*, zonula occludens 2.
